# Mesenchymal stroma cells in peritoneal dialysis effluents from patients

**DOI:** 10.1007/s13577-016-0155-5

**Published:** 2017-01-05

**Authors:** Bin Liu, Qiunong Guan, Jing Li, Gerald da Roza, Hao Wang, Caigan Du

**Affiliations:** 1 Department of Critical Care Medicine, Tianjin Medical University General Hospital, Tianjin, 300052 China; 2 Department of Urologic Sciences, University of British Columbia, Vancouver, BC Canada; 3 Department of Anesthesiology, General Hospital of Tianjin Medical University, Tianjin, 300052 China; 4 Fraser Health Nephrology, Royal Columbian Hospital, New Westminster, BC Canada; 5 Department of General Surgery, Tianjin Medical University General Hospital,154 Anshan Road, Heping District, Tianjin, 300052 China; 6 Jack Bell Research Centre, Room 250A, 2660 Oak Street, Vancouver, BC V6H 3Z6 Canada

**Keywords:** Peritoneal cavity, Peritoneal dialysis effluent, Peritoneal mesenchymal stroma cells

## Abstract

Mesenchymal stroma cells **(**MSCs) have potential as an emerging cell therapy for treating many different diseases, but discovery of the practical sources of MSCs is needed for the large-scale clinical application of this therapy. This study was to identify MSCs in peritoneal dialysis (PD) effluents that were discarded after PD. The effluents were collected from patients who were on the dialysis for less than 1 month. Adherent cells from the effluents were isolated by incubation in serum-containing medium in plastic culture dishes. Cell surface markers were determined by a flow cytometric analysis, and the in vitro differentiation to chondrocytes, osteocytes or adipocytes was confirmed by staining with a specific dye. After four passages, these isolated cells displayed the typical morphology of mesenchymal cells in traditional 2-D cultures, and were grown to form spherical colonies in 3-D collagen cultures. Flow cytometric analysis revealed that the unsorted cells from all of seven patient samples showed robust expression of typical mesenchymal marker CD29, CD44, CD73, CD90 and CD166, and the absence of CD34, CD79a, CD105, CD271, SSEA-4, Stro-1 and HLA-DR. In differentiation assays, these cells were induced in vitro to chondrocytes, osteocytes or adipocytes. In conclusion, this preliminary study suggests the presence of MSCs in the “discarded” PD effluents. Further characterization of the phenotypes of these MSCs and evaluation of their therapeutic potential, particularly for the prevention of PD failure, are needed.

## Introduction

Mesenchymal stroma cells **(**MSCs), historically termed as mesenchymal stem cells, are multipotent fibroblast-like, adult stroma cells that were first isolated from the bone marrow (BM) [1], and have been recognized capacity for both self-renewal and multilineage differentiation potentials [2, 3]. Due to their multipotency and paracrine effects [4, 5], MSCs become ideal candidates for cell therapy in regenerative medicine [6, 7], which has been received much attention recently and progressed at a pretty fast pace during the past few years [8].

However, a feasible or practical source for the large-scale clinical use of MSCs has not really been established yet. The BM has been used as a major source of MSCs for both basic and clinical studies since 1976 when they were first isolated from the BM by Friedenstein [1], but BM harvesting is a relatively invasive procedure and only preferentially performed in adults. Recently, human umbilical cord (UC)-derived MSCs (and especially Wharton’s jelly) has been demonstrated to be a viable clinical alternative to BM-MSCs due to relatively easy harvest procedure of UC-MSCs without harm to the baby or mother [9], but the availability of UC-MSCs is still an issue for large qualities needed in clinics as therapeutic cells. Peritoneal dialysis (PD) is one of renal replacement treatments for individuals with kidney dysfunction, in which a PD solution is infused into the abdomen, followed by drainage out effluent containing patient’s fluid, cells and “waste”. The objective of this study was designed to examine the presence of MSCs in the “discarded” PD effluent to develop MSCs-based therapy at least for the prevention of ultrafiltration failure (UFF) or peritoneal membrane injury in PD patients.

## Materials and methods

### Collection of PD effluents

PD effluents were randomly collected from anonymized patients (both male and female, 45–66 years old) who were on PD therapy with either Dianeal or Physioneal PD solution within 4 weeks (Table 1). We were not able to isolate MSCs from the effluents of several clinically stable patients (data not included).

**Table 1:** The demographic information of donors

Donor number	Age (year)	Gender (F/M)	NIH ethnicity categories	Time on PD (week)	PD solution
1	57	M	White	3	Dianeal
2	51	F	Asian	1	Dianeal
3	66	M	Asian	The first PD	Dianeal
4	60	M	White	1	Physioneal
5	45	M	Asian	The first PD	Physioneal
6	63	M	White	4	Dianeal
7	56	F	Asian	The first PD	Dianeal

The PD effluents were collected under protocol H15-02466 approved by the Clinical Research Ethics Board at the University of British Columbia (H15-02466)

### Isolation and growth of PD effluent-derived adherent cells

Cells were pelleted from PD effluents by centrifugation at 2000 rpm at 10 °C for 10 min within 12 h after collection from the patients, followed by washing once with phosphate-buffered saline (PBS). The erythrocytes in isolated cells were removed by a brief incubation (~4 min) with lysis buffer (0.15 M NH_4_Cl, 1.0 mM KHCO_3_, 0.1 mM EDTA, pH 6.8). After washing again with PBS, the resultant cells were finally suspended and cultured in plastic culture dishes with Dulbecco’s modified Eagle’s medium/Ham’s nutrient mixture F12 (DMEM/F12) containing 10% fetal bovine serum (FBS) and 1% penicillin/streptomycin at 37 °C in a 5% CO_2_ atmosphere incubator. The non-adherent cells were removed with each passage of cell culture.

### Flow cytometric analysis of cell surface markers

After four passages (P4), the expression level of a panel of MSC surface markers was measured using fluorescence-activated cell sorting (FACS) analysis with a specific fluorescent-conjugated monoclonal antibody. The following fluorescent-conjugated monoclonal antibodies were used in this study: Rat allophycocyanin (APC) anti-human/mouse CD44 (clone IM7, *e*Bioscience, San Diego, CA, USA), APC mouse anti-human CD34 (clone 4H11, *e*Bioscience), fluorescein isothiocyanate (FITC) mouse anti-human Stro-1 (clone MOPC-104E, BioLegend, San Diego, CA, USA), phycoerythrin (PE) mouse anti-human CD146 (clone P1H12, BD Biosciences, Mississauga, ON, Canada), APC mouse anti-human CD29 (clone TS2/16, BioLegend), FITC mouse anti-human CD90 (Thy-1) (clone eBio5E10, *e*Bioscienc), FITC mouse anti-human HLA-DR (clone L243, *e*Bioscience), PE mouse anti-human CD79a (clone HM47, *e*Bioscience), PE mouse anti-human CD166 (ALCAM) (clone 3A6, *e*Bioscience), APC mouse anti-human CD14 (clone 61D3, *e*Bioscience), FITC mouse anti-human CD105 (Endoglin) (clone 266, BD Biosciences), APC mouse anti-human CD45 (clone H130, BD Biosciences), PE mouse anti-human CD271 (clone C40-1457, BD Biosciences), FITC mouse anti-SSEA-4 (clone MC813-70, BD Biosciences), and PE mouse anti-human CD73 (clone AD2, BD Biosciences). The single cell suspension was incubated with each type of the antibodies in the dark for 30 min at 4 °C. After washing with PBS, the fluorescence intensity of the stain was counted using a Calibur flow cytometer (BD Biosciences). Data were analyzed with FlowJo software (FlowJo, LLC., Ashland, OR, USA).

### Colony formation in 3-D cultures

After P4, adherent cells were grown in a 3-D collagen cell culture system (Catalog: ECM 675, Millipore-Canada, Etobicoke, ON, Canada) following manufacturer’s protocol. In brief, 200 µl of collagen gel solution (66%) was added to each well in 24-well plates, and was prepared by mixing 132 µL of collagen solution, 20 µl of 10 × PBS and 20 µl of 0.1 N NaOH with 28 µl of DMEM/F12 medium containing 2–5 × 10^3^ cells at 4 °C, followed by collagen polymerization at 37 °C for 2 h. The 3-D cultures were incubated with complete DMEM/F12 medium (containing 10% FBS) in a humidified 5% CO_2_ incubator at 37 °C. Colonies were observed under a light microscope after approximately 3 weeks of incubation.

### Multigenic differentiation

The chondrogenic differentiation was performed using a high-density cell culture. In brief, after P4 1 × 10^6^ cells in a volume of 10 μL were dropped on to a 10 cm petri dish and incubated at 37 °C for 2 h, followed by incubation in high glucose DMEM medium supplemented with 1% FBS 10 ng/mL transforming growth factor-β1 (Sigma, Germany), 50 μg/mL ascorbate acid, 0.1 μM dexamethasone, 100 μg/mL sodium pyruvate, 40 μg/mL proline and 50 mg/mL ITS premix (5 μg/mL insulin, 5 μg/mL transferrin and 5 ng/mL selenious acid). The cultures were maintained for 4 weeks, during which the medium was exchanged twice a week. At the end of the incubation period, the cells were stained with 1% of acidic Alcian blue in 80% methanol (v/v) (pH 2.5) to confirm chondrogenic differentiation or the presence of cartilage formation.

The osteogenic or adipogenic differentiation was induced in confluent cultures in plastic culture dishes after P4 according to the published protocol [10, 11]. For osteogenic differentiation, the cells (1 × 10^6^ cells/well in 6-well plates) were treated with high glucose DMEM supplemented with 10% FBS, 50 μg/ml ascorbic acid, 10 nM dexamethasone (Sigma-Aldrich Canada, Oakville, ON, Canada), 10 mM β-glycerol phosphate (Sigma-Aldrich Canada) and 3.7 mg/mL sodium bicarbonate for 4 weeks. During this period of incubation, the culture medium was exchanged twice a week, followed by staining with 2% Alizarin red S in 0.5% NH_4_OH (pH 4.2) to confirm the presence of Ca2^+^ matrix mineralization. For adipogenic differentiation, the cells (1 × 10^6^ cells/well in 6-well plates) were incubated with high glucose DMEM supplemented with 10% FBS, 5 nM hydrocortisone, 50 μg/mL ascorbic acid, 50 μg/mL indomethacin and 1 μM dexamethasone, and the culture medium was exchanged twice a week for 4 weeks. Adipogenesis was confirmed for the presence of lipid droplets stained with 0.14% Oil red O following the protocol in Lonza website (www.lonza.com).

## Results

### Cell morphology in 2- and 3-D cultures

**Fig. 1:**
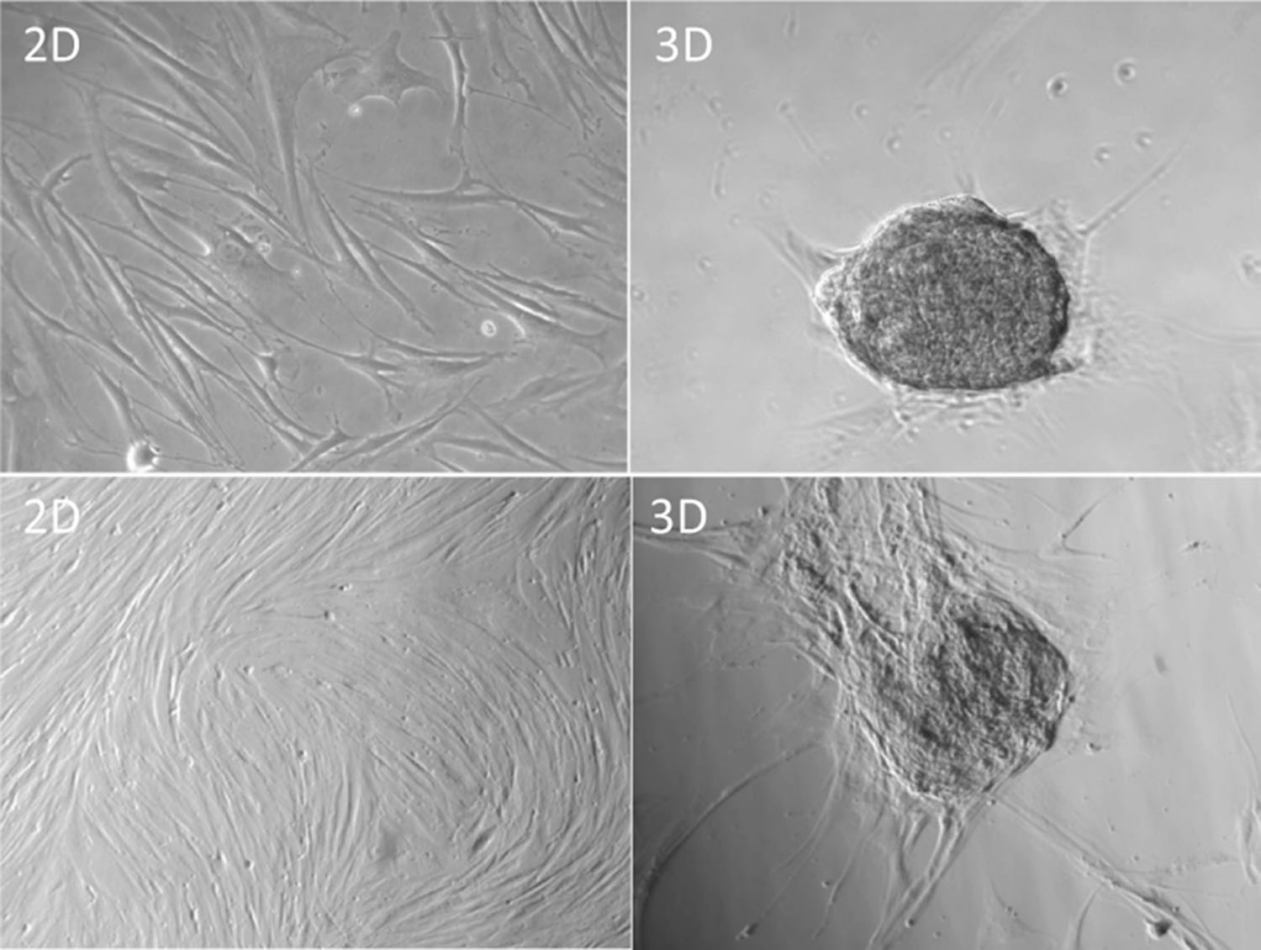
Morphology of PD effluent-derived adherent cells in cultures. Cells from PD effluents were grown in plastic culture plates. After four passages, unsorted adherent cells were grown in culture plates (2D) compared to those in 3-D collagen cell culture system (3-D). Data were presented as typical microscopic views of cultured cells—both non-confluence (*upper right*) and confluence (*bottom right*) of cells in 2-D cultures, and spherical colonies in 3-D cultures

The change in morphology of PD effluent-derived adherent cells in plastic culture dishes was observed microscopically at every passage, and non-adherent cells were removed by changing the medium each time. Adherent long spindle-shaped cells were presented at the second passage (2 weeks) after isolation. Later on the proportion of enlarged flat or thin, long spindle-shaped fibroblast-like cells with altered morphology gradually increased or propagated well in the culture dishes, which became obvious at the fourth passage (Fig. 1). The number of adherent cells varied from patient to patient, but was about 2.5 × 10^6^ cells in average recovered after P4 from one PD effluent sample (2 L volume) in this limited population of patients. Further experiments using a 3-D culture system showed that these fibroblast-like cells formed spherical colonies in collagen-based 3-D environment (Fig. 1).

### Cell surface markers

**Fig. 2:**
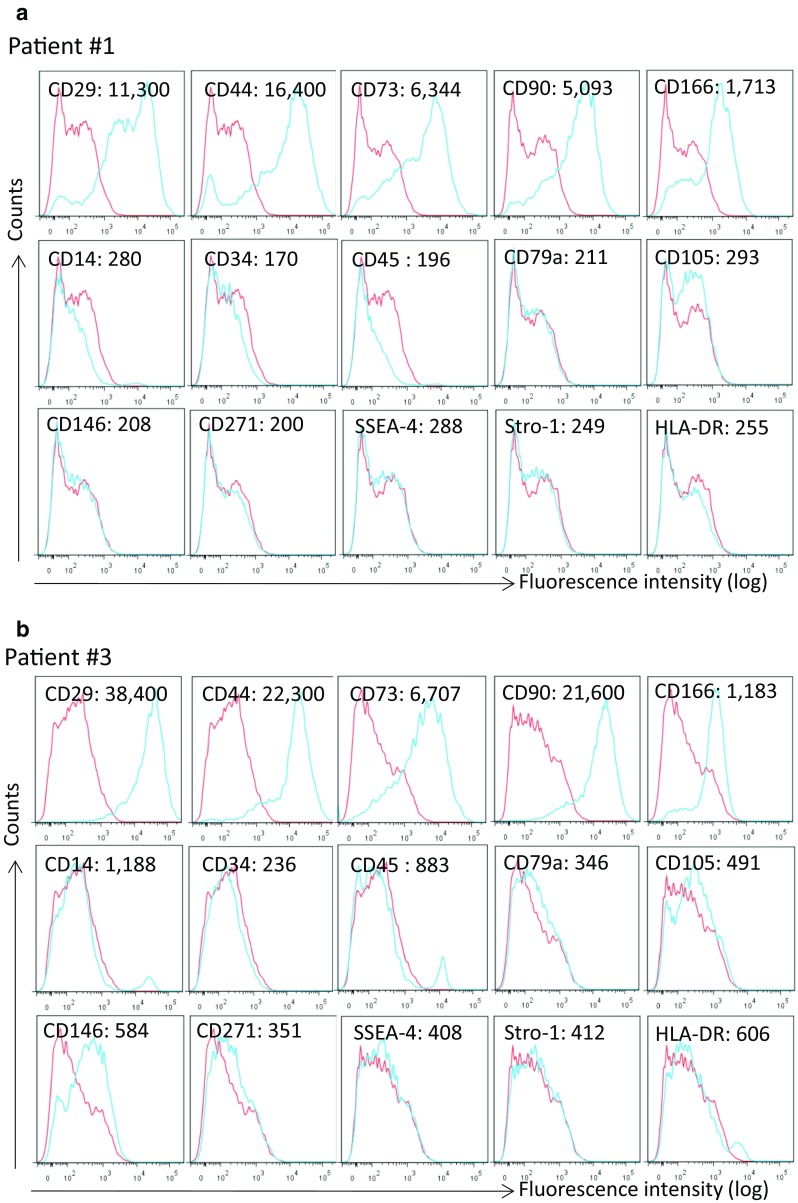
The expression of cell surface markers in PD effluent-derived adherent cells after four passages. Cells from PD effluents were grown in plastic culture plates. After four passages, the expression of cell surface markers in unsorted cells was analyzed using FACS analysis, and was presented by the mean fluorescence intensity (MFI). Data were presented as typical FACS graphs from seven donors. **a** Donor #1. **b** Donor #3. *Blue line* fluorescence intensity of anti-target marker antibody, *red line* background fluorescence intensity of control antibody

Flow cytometry analysis revealed that after P4 PD effluent-derived adherent cells from all of seven donors were absolutely positive in the expression of CD29, CD44, CD73, CD 90, and CD166, and negative in CD34, CD79a, CD105, CD271, SSEA-4, Stro-1 and HLA-DR (Fig. 2; Table 2). The expression of other three markers (CD14, CD45 and CD 146) among these donors was heterogenic, indicated by the fact that a small peak of high fluorescence intensity (>10^4^) in the staining of both CD14 and CD45 was seen in the unsorted cells from donor #3, #4 and #7 (Fig. 2b; Table 2), which might imply that there had a small proportion of the cells from these donors expressed CD14 and/or CD45. As compared to the background staining (MFI: 229), the higher levels of MFI (422–584) were seen in CD146 staining of the cells from donor #2 to #6 (except of #1 and #7) (Table 2), suggesting that the unsorted cell population from these donors might weakly express this marker.

**Table 2:** The expression (mean fluorescence intensity: MFI) of cell surface markers in PD effluent-derived adherent cells from each donor after 4 passages in culture

Antibody	1	2	3	4	5	6	7
CD14-APC	280	167	1188	843	586	392	1316
CD29-APC	11,300	35,400	38,400	43,100	6105	3683	14,200
CD34-APC	170	177	236	356	252	354	146
CD44-APC	16,400	15,800	22,300	34,300	23,700	2132	7255
CD45-APC	196	148	883	500	309	317	421
CD73-PE	6344	11,300	6707	12,200	6728	3890	8430
CD79-PE	211	502	346	438	421	401	234
CD90-FITC	5093	24,500	21,600	9402	10,800	10,900	4672
CD105-FITC	293	803	491	508	251	414	217
CD146-PE	208	548	584	556	501	422	195
CD166-PE	1713	1761	1183	878	1455	922	145
CD271-FITC	200	549	351	407	458	432	230
HLA-DR-FITC	255	292	606	446	226	360	382
SSEA-FITC	288	234	408	475	205	367	171
Stro-1-FITC	249	293	412	469	212	333	193

Data were presented by MFI. Background MFI of staining with each fluorescence dye (*n* = 7): 236.14 ± 60.08 [mean ± standard derivation (SD)] of APC, 334 ± 70.7 of PE and 294 ± 79.51 of FITC

*APC* allophycocyanin, *PE* phycoerythrin, *FITC* fluorescein isothiocyanate

### Multipotential for differentiation to chondrocytes, osteocytes or adipocytes

**Fig. 3:**
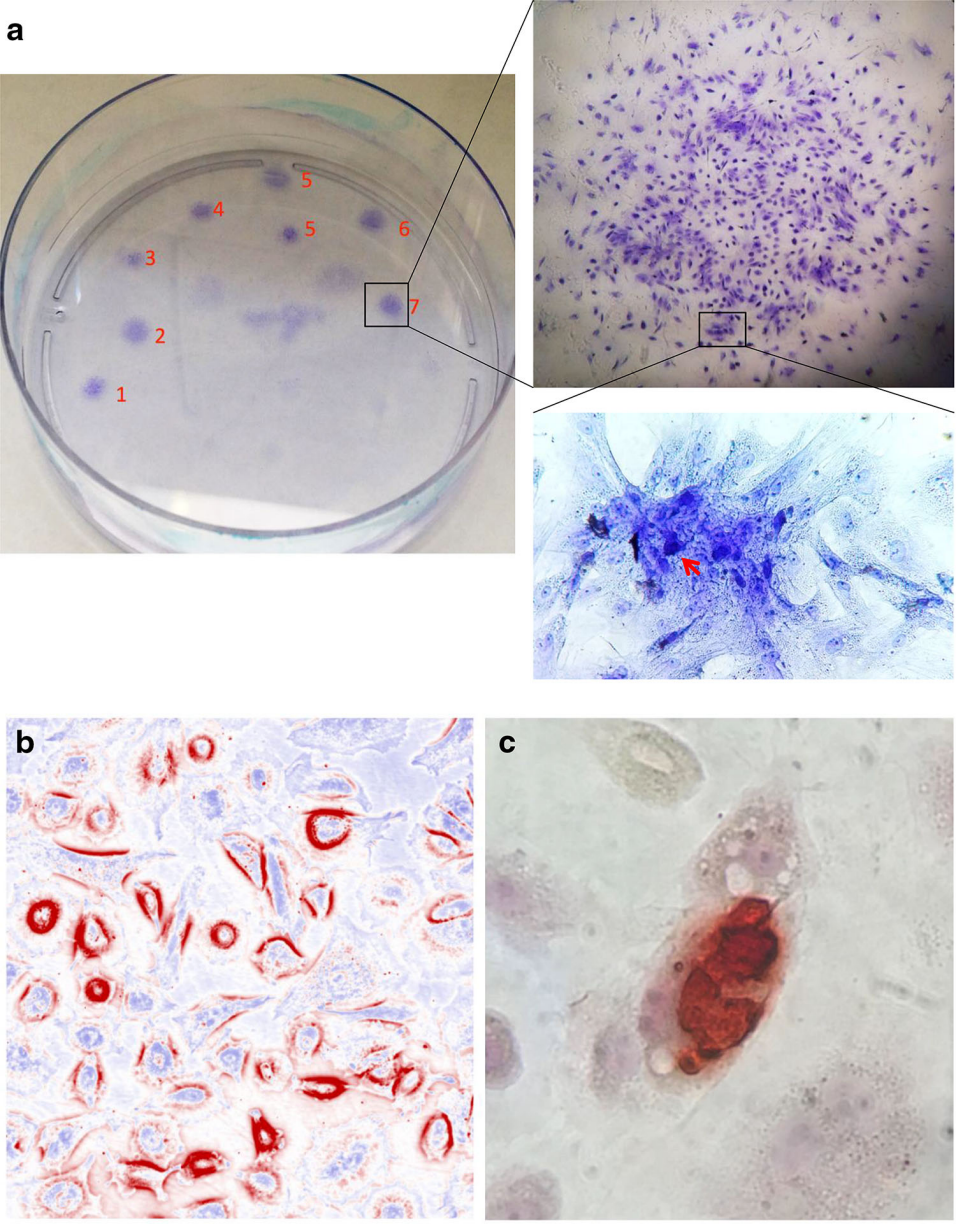
Multipotency of PD effluent-derived adherent cells in cell differentiation. Cells from PD effluents were grown in plastic culture plates. After four passages, the unsorted cells were induced to chondrocytes, osteocytes or adipocytes by incubation with each different medium for 4 weeks. **a** Differentiation to chondrocytes. All seven donor samples were incubated with chondrogenic medium in the same plate (labeled by the donor number), and were stained with Alcian blue. *Arrow* cartilage matrix in “clustering” cells. Data were a representative of three separate experiments. **b** Differentiation to osteocytes. A confluent monolayer of PD effluent-derived adherent cells was incubated with osteogenic medium, and was stained with Alizarin red S. *Red* extracellular calcium deposits. Data were presented as a typical microscopic view of seven donor samples. **c** Differentiation to adipocytes. A confluent monolayer of PD effluent-derived adherent cells was incubated with adipogenic medium, and was stained with Oil* red* O. *Red* lipid droplets. Data were presented as a typical microscopic view of positive stain of lipid droplets inside the cells

After P4, the potential for differentiation of PD effluent-derived adherent cells to chondrocytes, osteocytes or adipocytes was examined separately in vitro. As shown in Fig. 3a, the PD effluent-derived cells from all of 7 donors were differentiated to the phenotype of chondrocytes, indicated by the presence of Alcian blue-stained cartilage matrix in “clustering” cells. Similarly, the differentiation of a confluent monolayer of these cells to osteocytes or adipocytes was seen after incubation with each different differentiation medium for 4 weeks, in which the presence of extracellular calcium deposits in osteocytes were confirmed by alizarin red S staining (Fig. 3b), and the lipid droplets in some of differentiated cells (adipocytes) by oil red O-staining (Fig. 3c). In control cultures with complete DMEM/F12 medium, the staining with all of these three dyes was negative (data not shown).

## Discussion

Although human MSCs have been recently receiving much attention for their potential in various therapeutic approaches [12], particularly for tissue regeneration [13] and immunomodulation [14], the source of MSCs for the large-scale clinical application of this cell therapy is not available. The BM and adipose tissues are the two main sources for both the experimental and clinic researches; however, accumulating evidence has shown several of their shortcomings, including the invasive procedure for sample collection, age-dependent differentiation potential and decreased proliferation, which may limit their extensive applicability [15]. Thus, it is of importance to find alternative sources of MSCs to overcome the above key limitations in this field.

In general, MSCs can be isolated from other types of cells and characterized by their morphology of fibroblast-like and adhering to plastic in 2-D cultures [2], and by their formation of spherical colonies in 3-D culturing environments [16, 17]. In addition to that, human MSCs, such as from the BM, have been demonstrated to have potential of multilineage differentiation to mesenchymal tissues, especially to the chondrocytes, osteocytes and adipocytes [2, 18, 19]. Similarly, in this study fibroblast-like cells were isolated from PD effluents by their adherence to the plastic and formed spherical colonies inside collagen-based matrix (Fig. 1), and in vitro these PD effluent-derived adherent cells could be differentiate to the chondrocytes, osteocytes and adipocytes (Fig. 3), suggesting that some of these PD effluent-derived cells (probably a subpopulation of them) have similar characteristics of adult MSCs isolated from BM.

Currently, there is no consensus on a single surface molecule to identify human MSCs from various sources, but the expression of several cell surface proteins has facilitated their identification or enrichment in the literature: Human MSCs express cell surface markers, including CD10, CD13, CD29, CD44, CD90, CD49a-f, CD51, CD73 (SH3), CD105 (SH2), CD106, CD166 and Stro-1, and lack of expression of CD45, CD34, CD14 or CD11b, CD79a or CD19 and HLA-DR surface molecules [20, 21]. Other markers, such as CD151, CD271, stage-specific embryonic antigen-4 (SSEA-4), CD146, TROP2 and SUSD2, are also used as markers to sort MSCs [22]. However, whether these markers can be applied to every other source of MSCs, remains unknown. In this study, we examined the expression of the most of these cell surface markers in unsorted adherent cells from PD effluents after P4 in cultures, and similar to BM-derived MSCs, our samples from all of seven donors were highly expressing typical mesenchymal markers CD29, CD44, CD73, CD90 and CD166, and were negative in the expression of CD34, CD79a and HLA-DR (Table 2; Fig. 2). In this study, we did not investigate the initial number of MSCs in the PD effluents prior to culturing in the plastic dishes. However, evidence in the literature suggests that the dialysate cells in PD effluents are largely composed of leukocytes (81%), erythroid lineage (14%) and a small portion of hematopoietic stem cells (Lin^−^/CD34^+^/CD38^−^/CD90^+^ phenotype, approximately 0.14 ± 0.03%) [23], and include peritoneal mesothelial cells as well [24], suggesting that MSCs are a very small portion of the dialysate cells in the PD effluents without in vitro enrichment.

There were some differences between PD effluent-derived cells and BM-MSCs; the PD-derived adherent cells from this limited number of donors were negative in the expression of CD271, Stro-1 and SSEA-4, and weakly positive in hematopoietic marker CD14 and CD45 in some of these samples (Fig. 2; Table 2). SSEA-4 is an early embryonic glycolipid antigen for undifferentiated pluripotent human embryonic stem cells and has been identified as a marker for the MSCs from BM and other tissues [25–27], but recent studies have reported that the expression of SSEA-4 is not necessary for the multipotency of many types of MSCs, such as from Wharton’s jelly and synovium [27–29]. Similar to SSEA-4, CD271 has been demonstrated to have different roles in MSCs from different tissues, such as BM versus UC or Wharton’s jelly [30, 31], indicated by that CD271 is an efficient marker for the MSC isolation from the BM or adipose tissue but not from UC or others, suggesting that the role of this molecule varies from different tissues. Indeed, the PD effluent-derived adherent cells isolated in this preliminary study had multipotency of differentiation to all three chondrocytes, osteocytes and adipocytes and were also negative in both SSEA-4 and CD271. Surprisingly, Stro-1 has been demonstrated in the literature almost as a unique marker for cloning ability and multipotency of MSCs from all different tissues [22, 32–34], but was not found in the PD effluent adherent cells in this study, suggesting that this molecule just like CD271 and SSEA-4 may not be an essential marker for all of MSCs.

We included three typical hematopoietic markers (CD14, CD45 and HLA-DR) in the examination of cell surface markers of unsorted PD effluent-derived adherent cells after P4. A clear indication of positive staining of both CD14 and CD45 was seen in some samples, such as donor #3 and #10 (Fig. 2b; Table 2). It has been well known that these two molecules are highly expressing on the surface of monocytes and macrophages [35, 36], suggesting the possibility of the presence of monocytes/macrophages from donor’s peritoneal cavity or the blood circulation in some of unsorted PD-derived adherent cells. In addition, it has been noted that CD45 is found in MSCs isolated from testis biopsies, ovary, hair follicle and UC Wharton’s jelly [37], and CD14 at a low level in non-myeloid lineage cells [38], implying that these two molecules may express in some of this newly identified MSCs from some donors which, however, remains further investigation.

One limitation of this study was that we did not investigate the tissue origin of these MSCs in PD effluents. It has been documented that multipotent MSCs can be found in nearly every tissue, including BM, adipose tissue, muscle, tendon and circulating peripheral blood [39, 40]. When patients start PD therapy with a bioincompatible and hypertonic PD solution, tissue injury occurs around the peritoneum and is evidenced by the presence of peritoneal mesothelial cells in the PD fluid [24]. Thus, it is reasonable to presume that MSCs in the damaged peritoneum may be released to the PD effluents. In consistent with our data, a recent study shows that the peritoneal mesenchymal cells isolated from human peritoneal lavage fluid display some MSC surface markers (positive: CD90, CD73, CD105, CD166; negative: CD14 and CD45) [41], suggesting that the MSCs we identified in PD effluents may also come from the peritoneal lavage fluids. The tissue origin of these PD effluent-derived MSCs remains further investigation.

In conclusion, this preliminary study for the first time has described the presence of MSCs in the PD effluents that express essential MSCs markers (positive: CD29, CD44, CD73, CD90 and CD166; negative: CD34, CD79a and HLA-DR), and have the multipotential differentiation to the chondrocytes, osteocytes and adipocytes, suggesting that the “discarded” PD effluents probably can provide a practical source of MSCs for the large-scale clinical application due to a large pool of PD patients. Interestingly, several experimental studies have already demonstrated that intraperitoneally injection of MSCs is able to attenuate peritoneal inflammation and fibrosis in rodent models of peritoneal dialysis [42–44], indicating the possibility of using PD effluent-derived MSCs to reduce peritoneal membrane injury and fibrosis in PD patients.
